# Ginkgolic Acid Inhibits Coronavirus Strain 229E Infection of Human Epithelial Lung Cells

**DOI:** 10.3390/ph14100980

**Published:** 2021-09-26

**Authors:** Maimoona S. Bhutta, Daniel G. Sausen, Elisa S. Gallo, Harel Dahari, Gustavo F. Doncel, Ronen Borenstein

**Affiliations:** 1 Department of Microbiology and Molecular Cell Biology, Eastern Virginia Medical School, Norfolk, VA 23507, USA; BhuttaM@EVMS.EDU (M.S.B.); SausenDG@EVMS.EDU (D.G.S.); 2 Rush University Medical Center—Pinnacle Dermatology, Barrington, IL 60010, USA; 3 The Program for Experimental and Theoretical Modeling, Division of Hepatology, Department of Medicine, Stritch School of Medicine, Loyola University Chicago, Maywood, IL 60153, USA; hdahari@luc.edu; 4 CONRAD, Department of Obstetrics and Gynecology, Eastern Virginia Medical School, Norfolk, VA 23507, USA; DoncelGF@EVMS.EDU

**Keywords:** ginkgolic acid, coronavirus, antiviral, fusion inhibitor, HCoV-229E

## Abstract

Since December 2019, the COVID-19 pandemic has affected more than 200 million individuals around the globe and caused millions of deaths. Although there are now multiple vaccines for SARS-CoV-2, their efficacy may be limited by current and future viral mutations. Therefore, effective antiviral compounds are an essential component to win the battle against the family of coronaviruses. Ginkgolic Acid (GA) is a pan-antiviral molecule with proven effective in vitro and in vivo activity. We previously demonstrated that GA inhibits Herpes Simplex Virus 1 (HSV-1) by disrupting viral structure, blocking fusion, and inhibiting viral protein synthesis. Additionally, we reported that GA displays broad-spectrum fusion inhibition encompassing all three classes of fusion proteins, including those of HIV, Ebola, influenza A, and Epstein Barr virus. Here, we report that GA exhibited potent antiviral activity against Human Coronavirus strain 229E (HCoV-229E) infection of human epithelial lung cells (MRC-5). GA significantly reduced progeny virus production, expression of viral proteins, and cytopathic effects (CPE). Furthermore, GA significantly inhibited HCoV-229E even when added post-infection. In light of our findings and the similarities of this family of viruses, GA holds promising potential as an effective antiviral treatment for SARS-CoV-2.

## 1. Introduction

In December 2019, the first case of Severe Acute Respiratory Syndrome Coronavirus 2 (SARS-CoV-2) was reported in Wuhan, China. SARS-CoV-2 was rapidly recognized as a global pandemic (termed COVID-19) by the World Health Organization [1,2].

Coronavirus (CoV) is a single-stranded, enveloped virus with a positive-sense RNA genome (27-to-32kb). Spike (S) proteins, which project from the enveloped surface, are classified as class I viral fusion proteins. S proteins form a homotrimer, which is cleaved by host proteases into S1 and S2 subunits, both important in cell entry. S1 protein is responsible for receptor binding, and S2 protein plays a crucial role in membrane fusion [3,4]. Human CoV (HCoV) falls under two genera, *alphacoronavirus* (HCoV-229E and HCoV-NL63) and *betacoronavirus* (HCoV-HKU1, HCoV-OC43, Middle East Respiratory Syndrome Coronavirus (MERS-CoV), SARS-CoV, and SARS-CoV-2) [3].

According to the John Hopkins Coronavirus Resource Center, more than 200 million COVID-19 cases have been reported globally as of August 2021 with a death toll greater than 4,000,000 people and rising. The United States accounts for >37,000,000 confirmed cases and >600,000 deaths [5]. Following an increase in global travel, the USA has experienced another surge of COVID-19 cases caused by variants of SARS-CoV-2 isolated from patients in the United Kingdom (B.1.1.7 alpha variant), South Africa (B.1.351 beta variant), and Brazil (P.1 or 501Y.V3 gamma variant) [5,6,7]. However, most of the newly confirmed cases are attributed to the recently discovered B.1.617.2 delta variant. The delta variant was identified in October 2020 in India. It was reported to contain mutations in the N-terminal domain and the receptor-binding domain of the S protein, thus increasing its immune evasion properties. According to recent literature, the effectiveness of single and double doses of the AstraZeneca or Pfizer vaccine was considerably lower for the Delta variant, with the double dose effectiveness estimated to be 60 and 88%, respectively [6,8]. Although current evidence indicates that the immune response generated by the available vaccines is robust for certain SARS-CoV-2 variants, the effectiveness of long-term immunity remains to be determined. Moreover, vaccine hesitancy and lack of vaccine availability aid in the global dissemination of SARS-CoV-2; hence, there is a growing need for therapeutic agents to mitigate the pathogenesis of the rapidly mutating variants, thereby minimizing morbidity and mortality [9].

*Ginkgo biloba* is a gymnosperm species used in Chinese herbal medicine since the 16th century. The seeds of *G. Biloba* were used as medicinal materials in traditional Chinese medicine for skin-based ailments as documented in ‘*The Compendium of Materia Medica Ben Cao Gang Mu*’ [10]. In the recent century, pharmaceutical interest has focused on the properties of *G. Biloba* extracts for the treatment of vascular disorders, vertigo, tinnitus, and various cognitive disorders [11,12]. Nowadays, *G. Biloba* is mainly present in East Asian countries such as China, Korea, and Japan [13]. *G. Biloba* is comprised of terpene trilactones (ginkgolides A, B, C, J), flavonols, glycosides, biflavones, proanthocyanidins, simple phenolic acids, 6-hydroxykynurenic acid, 4-O-methylpyridoxine, polyprenols, and alkylphenols [11,12,13]. Ginkgolic acid (GA) is an alkylphenol constituent extracted from the leaves and seed coats of *G. biloba* [10,14,15,16]. Previous literature has shown that GA demonstrates pleiotropic bioactive properties, including anti-inflammatory [17], antitumor [15,18], and antibacterial [19] effects. Our lab has recently shown that GA has antiviral activity against enveloped viruses such as Herpes Simplex Virus 1 (HSV-1), Human Cytomegalovirus (HCMV), and Zika Virus (ZIKV) through inhibition of viral fusion. GA has also shown a broad spectrum of fusion inhibition encompassing all three classes of fusion proteins, indicating that GA inhibits virion entry by blocking the initial fusion event [20,21]. We herein report that GA has a dose-dependent antiviral effect against human *alphacoronavirus* strain 229E (HCoV-229E) infection of human MRC-5 lung fibroblast cells. We also show that GA significantly inhibited HCoV-229E, even when added post-infection.

## 2. Results

### 2.1. GA Demonstrates Antiviral Activity against HCoV-229E in a Dose-Dependent Manner

**Figure 1:**
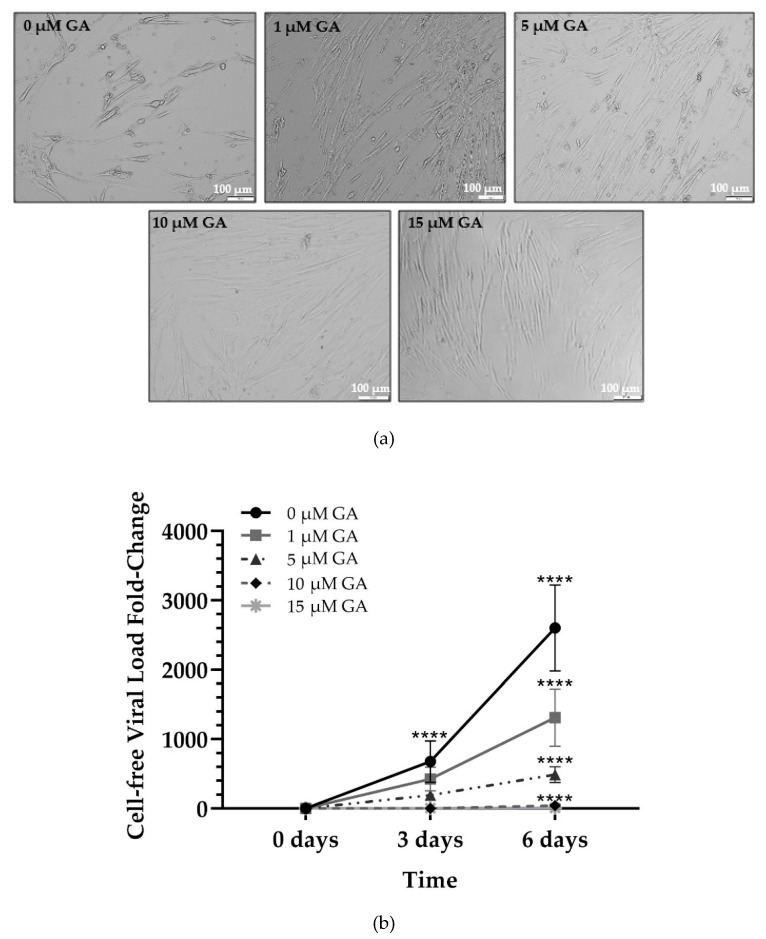

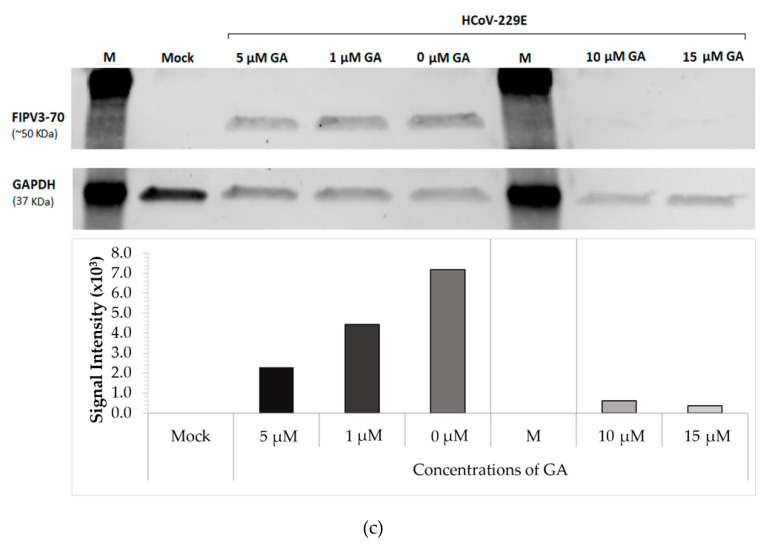
Dose-dependent response of GA in HCoV-229E-infected MRC-5 cells. (**a**) Representative light microscopy images of cytopathic effect (CPE) caused by HCoV-229E-infected MRC-5 cells treated with respective concentrations of GA on day 6 post-infection (pi); (**b**) supernatant viral load of infected MRC-5 cells treated with increasing concentrations of GA across the indicated time points, compared to day 0; (**c**) Western Blot analysis using antibodies directed against coronavirus (FIPV3-70); M: Chameleon 800 Pre-stained Protein Ladder. Band signaling intensities for FIPV3-70 antibody have been normalized to GAPDH. Mixed-model ANOVA [Interaction (Time*Treatment): [*p* < 0.0001]; treatments (0, 1, 5, 10, 15 µM GA): [*p* < 0.0001]; and time (0, 3, 6 days): [*p* < 0.0001]). **** *p* < 0.0001, represent a significant decrease in viral load under each concentration compared to 0 µM GA at day 6 pi.

Following our previous findings [20,21], we tested GA antiviral activity against HCoV-229E infection of MRC-5 cells in concentrations ranging from 0 to 15 µM. Confluent MRC-5 cells (75–85%) were pretreated with increasing concentrations of GA for 1 h at 37 °C. Following incubation, MRC-5 cells were infected with HCoV-229E at a MOI of 0.16 in the presence of GA for 2 h at 37 °C to allow the viral particles to enter the cell. After a 2 h incubation period, the culture media was aspirated, and fresh media with specific GA concentrations was added for six days at 33 °C. Samples were collected 2 h post-infection (p.i.) on day 0 (viral load in the aformentioned aspirated media), day 3, and day 6. The results indicated that GA inhibited HCoV-229E in a dose-dependent manner. Examining the phenotypical properties of the MRC-5 cells using a light microscope indicated that cells pretreated with higher concentrations of GA (10 and 15 µM) display a significantly decreased virus-induced cytopathic effect across six days of infection when compared to cells treated with no or lower concentrations of GA (Figure 1a). To examine GA’s antiviral effect, the relative levels of viral RNA were determined from cell culture supernatant at days 0, 3, and 6 p.i. As shown in Figure 1b, with increasing concentrations of GA a significant decrease in the viral fold change in comparison to cells treated with 0 µM GA (DMSO-control) was seen. A significant difference in the mean viral fold-change was found among the 3- and 6-day time points (*p* < 0.0001) across all GA concentrations (*p* < 0.0001), and when examining the interactions between time and concentration (*p* < 0.0001). Cells treated with 0 µM GA demonstrated 600-fold increase in viral load on day 3 p.i. compared to day 0. By day 3 p.i., cells treated with 1 µM exhibited a 37.3% decrease in fold change when compared with cells treated with 0 µM GA (DMSO only) (*p* = 0.0471). Similarly, cells treated with 5, 10, and 15 µM GA demonstrated a 71.6 (ns), 99.6 (ns), and 99.48% (ns) decrease at day 3 p.i., respectively, in viral load fold change when compared to 0 µM GA-treated cells. In contrast, cells treated with 0 µM GA had shown a 2600-fold increase in viral load on day 6 compared to day 0 (*p* < 0.0001). Cells treated with 1 µM exhibited a 49.6% decrease (*p* = 0.0004) in fold change when compared with cells treated with 0 µM GA on day 6. Similarly, by day 6, cells treated with 5, 10, and 15 µM GA demonstrated an 81.2% (*p* < 0.0001), 98.4% (*p* < 0.0001), and 99.7% (*p* < 0.0001) decrease, respectively, in viral load fold change when compared to 0 µM GA-treated cells. The results indicate that GA reduces viral progeny production in MRC-5 cells in a dose-dependent manner, with viral production significantly inhibited at each time point at all doses. Inhibition was particularly prominent following treatment with 10 and 15 µM GA. To verify our findings, we examined the expression of the HCoV-229E nucleoprotein, FIPV3-70, in infected MRC-5 cells using Western Blot analysis. As shown in Figure 1c, viral protein expression was completely diminished in cells treated with 10 or 15 µM GA. These findings suggest that GA fully abbrogates the expression of viral N protein at 10 and 15 µM concentrations.

### 2.2. Administration of GA Protects MRC-5 Cells Post-Infection

**Figure 2:**
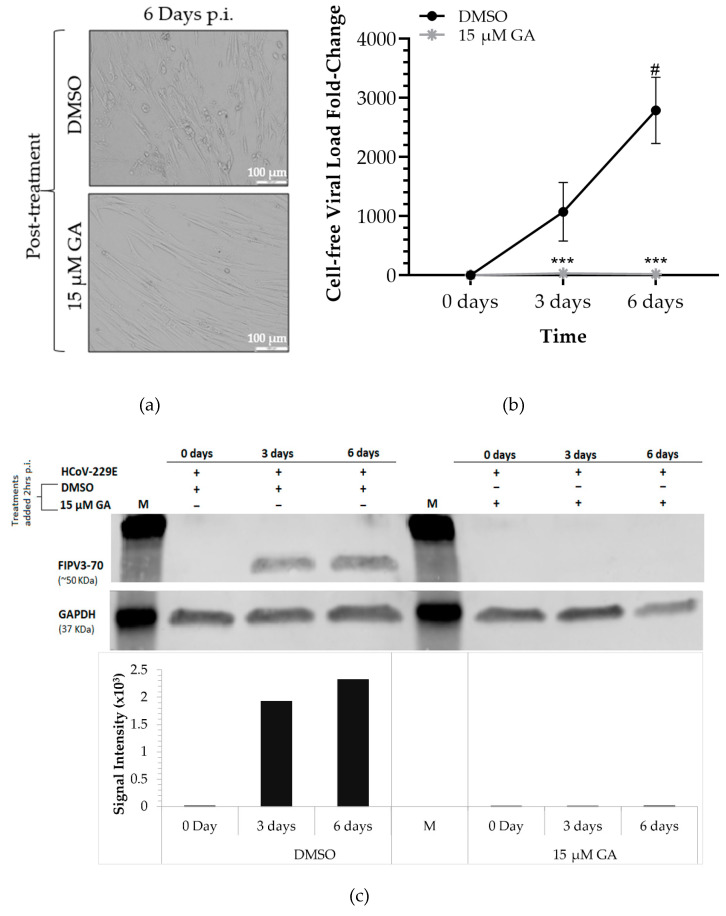
Post-treatment of Ginkgolic acid in HCoV-229E-infected MRC-5 cells. (**a**) Representative light microscopy images obtained on day 6 of MRC-5 cells treated post-infection with DMSO or 15 µM GA; (**b**) viral load fold-change detected from the supernatant of infected MRC-5 cells, post-treated with DMSO or 15 µM GA, indicated time points compared to day 0; (**c**) Western Blot analysis of cells treated post-infection using antibodies directed against coronaviruses and GAPDH. M: Chameleon 800 Pre-stained Protein Ladder. Signaling intensities of bands for FIPV3-70 antibody have been normalized to GAPDH. Mixed-model ANOVA of post-treatment experiments [Interaction (Time*Treatment): [*p* < 0.01]; Treatments (DMSO or 15 µM GA): [*p* < 0.001], and Time (0, 3, 6 days p.i.): [*p* < 0.01]). # *p* < 0.001, represents the significant increase in viral load at day 6 p.i. under the respective treatment when compared to day 0; *** *p* < 0.001 represents a significant decrease in the supernatant viral load of cells treated with 15 µM GA compared to the DMSO-treated cells at days 3 and 6 p.i.

To examine whether GA retains its antiviral effect post-HCoV-229E infection, we infected 85% confluent MRC-5 cells with 0.16MOI of HCoV-229E for 2 h at 37 °C. The infected cells were washed and fresh media was added to the cell culture with DMSO or 15 µM GA. The treated cells were incubated for 1 h at 37 °C before being incubated at 33 °C for six days. Following the application of the respective treatments, cells and cell culture supernatants were collected on days 0, 3, and 6. As observed in pretreatment experiments, cells treated post-infection with 15 µM GA demonstrated substantial inhibition of CPE across six days when compared to cells treated with DMSO (Figure 2a). Likewise, GA-treated cells displayed a minor change in viral load fold change compared to DMSO-treated cells after HCoV-229E infection (Figure 2b). Cells treated with DMSO (0 µM GA) exhibited a 1000-fold increase in viral load on day 3 and 2788-fold increase in viral load on day 6 (*p* = 0.0003) when compared to day 0. The results also indicated a significant increase in viral production in DMSO-treated cells from day 3 to day 6 (*p* < 0.05). Cells treated with 15 µM GA demonstrated a 33-fold increase on day 3, which lowered to a 17-fold change on day 6 compared to samples collected on day 0. Cells treated with 15 µM GA exhibited a 96.89% decrease in viral load on day 3 (*p* = 0.0004) and 99.37% decrease in viral load on day 6 (*p* = 0.0003) when compared to DMSO-treated cells at each respective time point. To verify our findings, we examined viral protein expression in MRC-5 cells using Western Blot analysis. As shown in Figure 2c, the expression of N protein was completely diminished (normalized to the signal intensity of GAPDH) in cells treated with 15 µM GA on days 3 and 6 post-infection when compared with DMSO-treated cells.

These results confirm that GA exhibits a protective antiviral effect against HCoV-229E. GA also protects human lung fibroblast cells following the infiltration of viral particles as it completely prevents secondary viral production and viral protein expression.

## 3. Discussion

Here, we report that GA in the range of 1 to 15 µM inhibits HCoV-229E CPE, viral RNA, and viral protein production in a dose-dependent manner. This effect is retained when cells are treated with 15 µM GA two hours post-infection with the virus. FIPV3-70 antibody binds to the nucleoprotein (N protein) of SARS-CoV-2 (~46kDa). The N protein is conserved among the alpha, beta, and gamma genera of coronaviruses, suggesting a common structural and functional role for N protein domains [22]. Furthermore, 15 µM GA completely inhibited coronavirus N protein expression on days 3 and 6 in post-infection treated MRC-5 cells. We previously reported that Ginkgolic acid’s antiviral mechanism of action involves inhibiting the initial virus-to-cell fusion event and preventing the spread of cell-to-cell infection [20]. In addition, we also reported that GA has broad-spectrum antiviral activity against all three classes of fusion proteins [20], which may explain its ability to inhibit coronavirus S protein, a class I fusion protein, which plays a role in cell entry [4]. Furthermore, our results also indicate that pre- or post-treatment with GA inhibits virus progeny production and protein expression evaluated on days 3 to 6 days post-infection. As we reported, GA inhibits the synthesis of viral proteins [16]; other researchers have also shown a potential secondary mechanism of action of GA involving inhibition of viral DNA and protein synthesis, which may explain the strong and successful inhibition of HCoV-229E and its potential to inhibit SARS-CoV-2 infection [15,17,18,23,24].

Increased degrees of severity of SARS-CoV-2 infections have been attributed to comorbidities (diabetes, hypertension, and obesity) arising from dysregulation, and thought to be compounded by an age-dependent decrease in metabolic processes [25,26,27]. Previous studies have revealed that GA inhibits de novo lipogenesis in pancreatic cancer (Panc-1), BxPC-3, and hepatocellular carcinoma (HepG2) cell lines by inducing the activation of 5′-adenosine monophosphate-activated protein kinase (AMPK) signaling and downregulating the expression of acetyl-CoA carboxylase (ACC) and fatty acid synthase (FASN) [11]. AMPK plays an essential role in the regulation of cellular energy metabolism. Coronavirus maintains a high ATP/AMP ratio following infection, reducing phosphorylation of AMPK (p-AMPK), AMPK substrates, and downstream targets. SARS-CoV-2 infection has been shown to influence components of the AMPK/mechanistic target of the rapamycin complex 1 (mTORC1) pathway. Downstream, coronaviruses activate autophagy inhibitors while reducing autophagy-enhancing proteins, proteins responsible for membrane nucleation, phagophore formation, and autophagosome-lysosome fusion [26,27]. GA has also been shown to activate AMPK in SW480 colon cancer cells and to decrease expression of invasion-associated proteins, including matrix metalloproteinase (MMP)-2, MMP-9, urinary-type plasminogen activator (uPa), and C-X-C chemokine receptor type 4 (CXCR4) in the SW480 cells. The effect of GA has been shown to be reversible following small interfering RNA (siRNA) silencing of AMPK expression, which suggests that GA can inhibit migration, invasion, and proliferation of colon cancer cells [18]. We propose that GA-induced activation of AMPK may hinder coronavirus propagation in primary human lung cells.

Liu et al. (2018) reported that GA directly binds and inhibits the SUMOylation E1 enzyme, thus inhibiting SUMOylation in both HEK293 and mBMSCs cells. SUMOylation is a post-translational modification by which small ubiquitin modifiers (SUMOs) are conjugated to protein targets by the E1, E2, and E3 sumoylation enzymes [24]. SUMOylation is known to play a role in cellular processes such as nuclear translocation, transcription regulation, apoptosis, stress response, protein stability, pluripotency, differentiation, and maintenance of stem/progenitor cells [24]. This has shown promise as a suppressor of cancer cell growth and migration [24]. Recent structural-based studies have examined posttranscriptional modifications of coronavirus proteins to understand the mechanism of virion assembly and virus-host interactions. The SUMOylation site in SARS-CoV was mapped to the lysine (62 amino acid) residue of the N protein [28]. N protein dimerizes and binds to genomic RNA, forming a nucleocapsid, which plays a significant role in viral genome replication and evasion of the immune response [3]. In SARS-CoV, post-transcriptional modification of N protein plays a vital regulatory role in viral replication cycles by interfering with host cell division [28]. Thus, we propose that the SUMOylation inhibition activity of GA may suppress post-transcriptional modification of the HCoV-229E conserved N protein, thereby disturbing the viral replication cycle.

The anti-inflammatory effects of GA and other Ginkgo Biloba extracts have been thoroughly examined in cell culture [29,30] and animal models [31,32]. GA significantly inhibits the production of NO, PGE2, proinflammatory cytokines (TNF-α, IL-1β, and IL-6) and suppresses the activation of iNOS and COX-2 in oxidized low-density lipoprotein (ox-LDL)-stimulated HUVEC cells. Application of GA in ox-LDL-induced HUVEC cells inhibits the degradation of IκB-α, preventing the translocation of NF-κB from the cytoplasm to the nucleus. GA also inhibits the phosphorylation of JNK, p38 MAPK, ERK, and Akt, thus strongly suppressing the activation of NF-κB [29]. The pathophysiology of SARS-CoV-2 resembles SARS-CoV infections. The onset of Acute Respiratory Distress Syndrome (ARDS) results from damage to the airways by aggressive inflammatory responses. Nuclear Factor Kappa B (NF-κB) is one of the major transcription factors activated in ARDS, and it plays a vital role in mediating immune responses to inflammation and other cellular activities. NF-κB activation can induce cytokine production, which leads to a positive autoregulatory loop that exacerbates the inflammatory response. 

Papain-like protease (PLP) of SARS-CoV has been shown to block phosphorylation and activation of interferon regulatory factor 3 (IRF3), thereby antagonizing interferon (IFN)-β induction [33,34]. Some reports have suggested that the ubiquitin-like domain of PLP is insufficient to block the activation of NF-κB, thus perpetuating the aggressive host inflammatory response to COVID-19 infection [33,34]. Thus, we postulate that GA could inhibit and suppress inflammation caused by severe SARS-CoV-2 infections by downregulating the expression of proinflammatory cytokines by suppressing the activation of the NF-κB signaling pathway. This approach should be tested in a relevant in vivo model of SARS-CoV-2 infection. A recent study using a high-throughput screen has identified GA and anacardic acid as irreversible inhibitors of SARS-CoV-2 PLP and 3-chymotrypsin-like protease (3-CLP) in Vero-E6 and BL21 (DE3) cells [35,36], thereby providing further evidence of the multifaceted role of GA as an antiviral inhibitor of coronaviruses.

## 4. Materials and Methods

### 4.1. Cell Line, Virus, and Compound

Human Coronavirus strain 229E (HCoV-229E, VR-740^™^, ATCC, Manassas, VA, USA) was propagated in the human lung fibroblast cell line MRC-5 (CCL-171, ATCC, Manassas, VA, USA). MRC-5 cells were maintained in ATCC-formulated Eagle’s Minimum Essential Medium (EMEM, Cat# 30-2003, ATCC, Manassas, VA, USA) supplemented with 10% heat-inactivated fetal bovine serum (FBS, Cat# 10082-147, Gibco, Rockford, IL, USA) and 1% penicillin and streptomycin (P/S, Cat# 15140-122, Gibco, Rockford, IL, USA). Viral titration of HCoV-229E (1 × 10^4^ TCID_50_/mL) was performed using a modified version of the protocol as previously described [37,38]. Next, 70–80% confluent MRC-5 cells were grown in a 96-well tissue culture plate. The cell culture media was aspirated, and cells were washed with 100 µL EMEM using a multichannel pipette. For the positive control, 100 µL of reference virus was added to the first row. The remaining wells were inoculated with serial logarithmic dilutions (corresponding dilution factors: 10^−1^-to-10^−7^) of the infected samples in EMEM supplemented with 1% (*v*/*v*) of FBS. The plate was incubated at 33 °C with 5% CO_2_ for 6 days, observing for endpoints in CPE. The medium was removed, and cells were delicately rinsed with Dulbecco’s phosphate buffered saline (0.01 M DPBS) (Cat# 14190-136, Thermo Fisher Scientific, Rockford, IL, USA). Cells were fixed with 100% methanol for 20 min at room temperature. The fixitive was removed, and the cells were stained with KaryoMax^®^ Giemsa stain (Cat# 10092-013, Gibco, Rockford, IL, USA). TCID_50_ was determined per 100 µL using the Reed and Muench method [34]. Ginkgolic Acid (GA, 50 mg) was purchased from Tocris (Cat# 6236, Minneapolis, MN, USA) and dissolved in solvent DMSO to generate a stock concentration of 100 mM. All experiments pertaining to HCoV-229E were conducting inside a biosafety hood of a BSL-2 laboratory at Eastern Virginia Medical School (Norfolk, VA, USA).

### 4.2. Treatments of HCoV-229E Infected MRC-5 Cells with Ginkgolic Acid

The dose-dependent response to GA was determined by preincubating MRC-5 cells in a 6-well tissue culture plate for 1 h on a shaker at 37 °C. GA concentrations included 0 µM (DMSO-control), 1, 5, 10, and 15 µM in EMEM media supplemented with 2% FBS and 1% P/S (EMEM/2%). Following incubation, the cells were infected with 0.16 MOI of HCoV-229E and left on the shaker for 2 h at 37 °C. After infection, the viral media was aspirated, cells were washed with 0.01 M DPBS, and fresh EMEM/2% media was added containing the respective concentrations of GA and day 0 samples (cells and cell culture supernatant) were collected. The infected cells were incubated for 6 days in 33 °C, and samples were taken on days 3 and 6.

GA treatment was also administered post-infection as MRC-5 cells were infected with 0.16MOI of HCoV-229E and left on the shaker for 2 h at 37 °C. Following incubation, the cells were washed with 0.01 M DPBS and fresh EMEM/2% media was added to the cells containing 15 µM GA or DMSO. The cells were incubated at 37 °C for 1 h on a shaker, then incubated at 33 °C for 6 days. Following the application of treatments, cells and cell culture supernatants were collected on days 0, 3, and 6.

### 4.3. qRT-PCR

Purified viral RNA was extracted from 140 µL of cell-free viral media using the QIAamp Viral RNA Mini Kit (Cat# 52906, Qiagen, Germantown, MD, USA) per the manufacturer’s instructions. The cDNA was then synthesized using iScript^™^ Reverse Transcription Supermix for RT-PCR (Cat# 1708841, Bio-Rad Laboratories, Hercules, CA, USA) per the manufacturer’s instructions. The cycling program consisted of initial priming for 5 min at 25 °C, followed by reverse transcription for 20 min at 46 °C, with RT inactivation for 1 min at 95 °C. The HCoV-229E primers and probe sequences listed in Table 1 were obtained from a previous publication [39].

The qPCR product was generated using the PrimeTime^®^ Gene Expression master mix (Cat# 1055772, IDT, Coralville, IA, USA) according to the manufacturer’s instructions. The reaction conditions were as follows: 1 min at 55 °C, 3 min at 95 °C, followed by 40 cycles of 15 s at 95 °C and 1 min at 53 °C. Reactions were performed in CFX96 Thermal cycler (Model# C1000 Touch, Bio-Rad, Hercules, CA, USA) and analyzed using CFX software (Version 3.1.1., Bio-Rad, Hercules, CA, USA).

### 4.4. Western Blot

Western blot analyses were conducted as previously described [20,21]. HCoV-229E-infected cells were washed twice with ice cold 0.01 M DBPS (Cat# 14190-136, Gibco, Grand Island, NY, USA) and collected using low speed centrifugation (3000 rpm for 5 min). Cells were lysed using Pierce^™^ RIPA buffer (Cat#89900, Thermo Fisher Scientific, Rockford, IL, USA) and Halt^™^ protease and phosphatase inhibitor cocktail (Cat# 78440, Thermo Fisher Scientific, Rockford, IL, USA) per the manufacturer’s instructions. Approximately 50 μg of proteins were electrophoretically separated on precast 4–15% Mini-Protean TGX Gels (Cat# 4561083, Bio-Rad Laboratories, Hercules, CA, USA) and transferred to 0.2 μm nitrocellulose membrane (Cat# 1704158, Bio-Rad Laboratories, Hercules, CA, USA). The membrane was blocked by Intercept^®^ Blocking Buffer (PBS; Cat# 927-70001, Li-Cor, Lincoln, NE, USA) for 20 min before applying primary antibodies overnight at 4 °C. The mouse monoclonal anti-coronavirus antibody (FIPV3-70; Cat# sc-65653, Santa Cruz Biotechnology, Dallas, TX, USA) was used at a dilution of 1:200. Membranes were washed four times with 0.01 M PBS Tween^™^−20 wash buffer (Cat# 28352, Thermo Fisher Scientific, Rockford, IL, USA) and then reacted with the appropriate IRDye^®^ 800CW Donkey anti-mouse secondary antibody (Cat# 926-32212, Li-Cor, Lincoln, NE, USA) at a dilution of 1:10,000, according to the manufacturer’s instructions. The membrane was stripped using NewBlot IR Stripping Buffer (Cat# 928-40028, Li-Cor, Lincoln, NE, USA) and then incubated with GAPDH-041 (Cat# sc-47724, Santa Cruz Biotechnology, Dallas, TX, USA) at a dilution of 1:200 overnight at 4 °C. The membrane was washed four times with 0.01 M PBS Tween^™^−20 wash buffer and then reacted with donkey antimouse secondary antibody. The membrane was analyzed using Odyssey CLx (Model# 9140, Li-Cor, Lincoln, NE, USA) and Image Analysis Software (Version 5.2.5., Li-Cor, Lincoln, NE, USA).

### 4.5. Statistical Analysis

Mixed model ANOVA with Tukey’s multiple comparisons test was used to analyze the viral load in the dose-dependent response experiments (independent experiments, n = 4) and post-treatment of MRC-5 cells with 0 µM GA (DMSO-control) or 15 µM GA (n = 3). In a dose-response experiment, unpaired t-tests were used to separately analyze the change in viral load between 0 µM GA and increasing GA concentrations on days 3 or 6, respectively.

## 5. Conclusions

We herein report GA as an inhibitor of the alphacoronavirus HCoV-229E. We have shown that GA inhibits CPE, viral RNA production, and viral N protein expression in infected human lung cells. MRC-5 cells pretreated with 10 to 15 µM GA demonstrate strong and effective antiviral activity in diminishing HCoV-229E viral load fold-change when compared with vehicle DMSO-treated cells. This effect is retained when cells were treated with GA two hours post-infection with HCoV-229E. The mechanism and pathways by which GA inhibits HCoV-229E are shared among other Coronaviruses. Therefore, GA holds promising potential to be an effective agent in treating SARS-CoV-2.

## Figures and Tables

**Table 1:** HCoV-229E primers with their respective sequences and melting temperatures.

Name	Melting Temperature	Sequences
Sense	54.9 °C	5′-GTTGTGGCCAATGGTGTTAAAG-3′
Antisense	55.2 °C	5′-AGTGTTGCCTGACTCTTTGG-3′
Probe	60.6 °C	/56-FAM/ACAATTTGCTGAGCTTGTGCCGTC/36-TAMSp/

## Data Availability

Data is contained within the article.
